# Assessing the Impact of Morphological Parameters on the Mechanical Behavior of Synthetic Meshes. A Multivariate Regression Approach

**DOI:** 10.1002/cnm.70092

**Published:** 2025-09-12

**Authors:** Vittoria Civilini, Alessandra Aldieri, Vincenzo Giacalone, Alberto L. Audenino, Mara Terzini

**Affiliations:** ^1^ Department of Mechanical and Aerospace Engineering Politecnico di Torino Turin Italy; ^2^ PolitoBIOMed Lab, Politecnico di Torino Turin Italy

**Keywords:** mechanical parameters, multivariate regression models, pores orientation, pores shape, synthetic meshes

## Abstract

The impact of morphological and mechanical parameters of surgical meshes on the healing processes and patient comfort after abdominal repair surgery is widely accepted. However, how the structure of the knitted pattern of synthetic meshes affects the mechanical behavior remains primarily theoretical. The objective of this study was therefore to assess the correlation between these key factors, identifying the most crucial morphological parameters able to support the design of new meshes. In this perspective, morphological parameters related to pore size, shape, and orientation were computed based on high‐resolution images using the *poreScanner* app and the Matlab Image Processing toolbox. Additional parameters such as weight and thickness were measured through high‐precision instruments. Concurrently, 12 mechanical parameters were assessed by executing a comprehensive testing protocol. Multivariate regression models were implemented, each using one to five morphological parameters as independent variables and one of the 12 mechanical parameters as dependent variables. A leave‐one‐out (LOO) validation algorithm was then employed to estimate the models' performance, robustness, and accuracy for potential future predictions. Regression models showed high coefficients of determination (*R* ^2^ ≥ 0.8), except for uniaxial strains (0.59 < *R* ^2^ < 0.71). The LOO validation reveals good predictive capabilities (*R* ^2^ > 0.65) for 5 out of 12 mechanical parameters, whereas moderate predictive capabilities (*R* ^2^ > 0.55) for one model. Promising results demonstrate a quantifiable relationship between pore characteristics and mechanical behavior. Thanks to further validation using different meshes, the models could be beneficial for all stakeholders involved in this field, from patients to manufacturers.

## Graphical abstract

A data‐driven regression framework was developed to quantify the relationship between mesh morphology and mechanical performance. Thickness, effective porosity, and pore orientation were consistently identified as key predictors, enabling accurate estimations and supporting improved strategies for surgical mesh design.

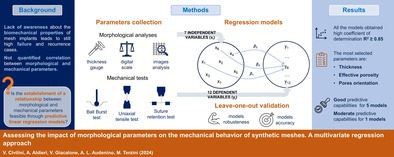

## 1 Introduction

**FIGURE 1:**
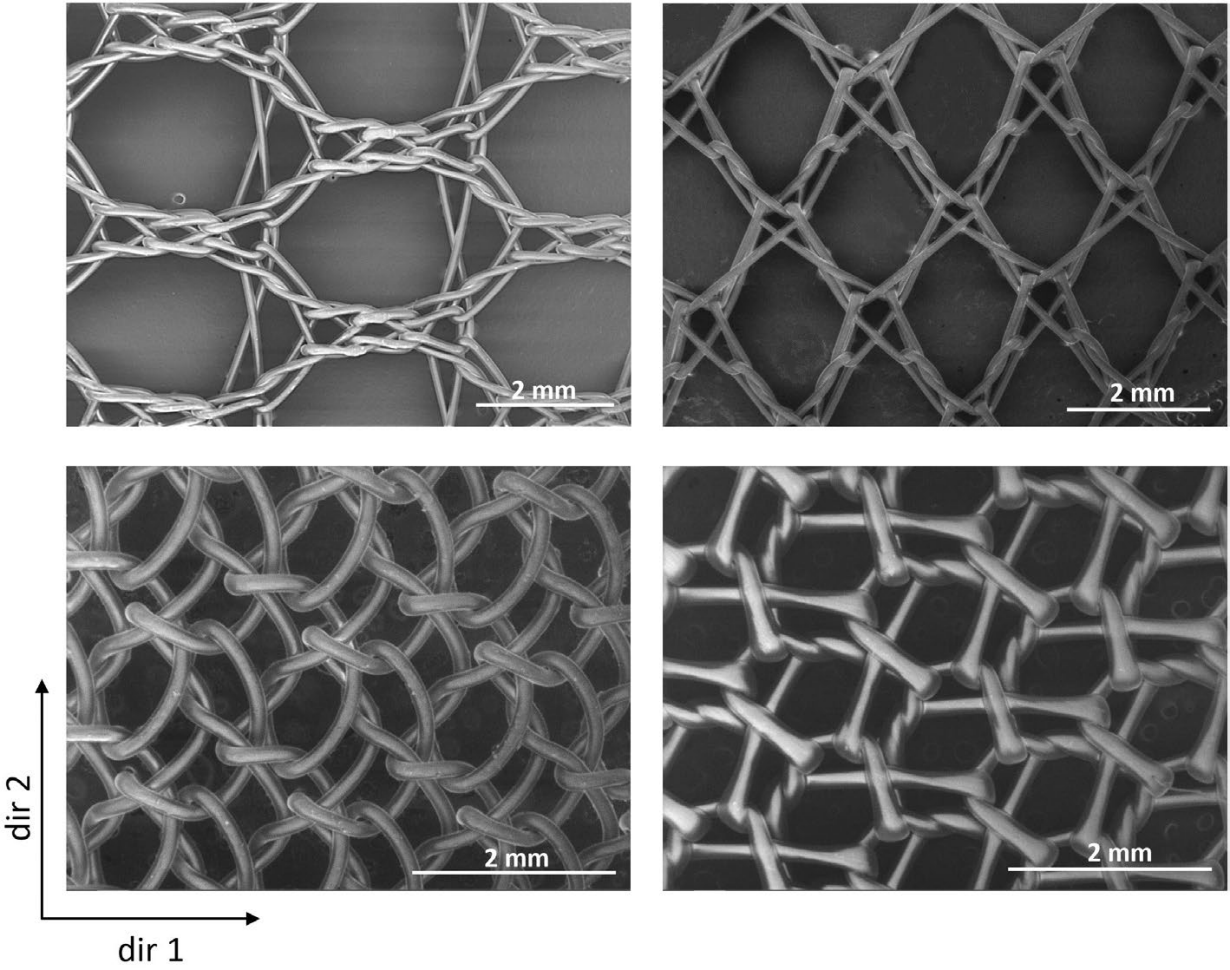
Knitted pattern of meshes with different densities. The two directions in the bottom left corner refer to the two principal directions of the knitted pattern.

At present, more than 70 meshes for hernia repair that differ in terms of material (synthetic, biologic, and composite) and manufacturing method are available on the global market [1]. Despite the introduction of many new materials, since the first implantation in 1958, polypropylene (PP) meshes are still the most implanted devices [2]. Most of the PP meshes currently available on the market are knitted, that is, comprised of continuous interlocking loops of yarns or threads. Changes in knitting patterns highly affect morphological parameters such as porosity, pore shape and orientation, as well as mesh thickness and weight [3]. This has led to the development of different devices characterized by various knitted patterns (Figure 1) that reflect different structures and consequently mechanical characteristics.

In accordance with the principles of functional tissue engineering, the ideal mesh should be able to be held in situ through peripheral sutures, promote a swift and well‐organized fibrous tissue response while minimizing inflammatory reactions, withstand biaxial tension loading and ensure minimal stiffness mismatch with the surrounding tissues [4]. However, since surgical meshes are implanted in a wide variety of body regions (e.g., abdominal region, pelvic region, groin region), they are subjected to various patterns of solicitations [5, 6, 7]. It is worthy of note that, nowadays, the occurrence of recurrences following the implantation of surgical meshes for hernia treatment falls within a range of 1.4% to 26.5% [8]. Hernia repair failures are often attributed to factors such as foreign‐body reaction and mesh shrinkage caused by incorrect tissue ingrowth during the healing process [9]. In this context, different and often contrasting opinions have arisen over the years about the morphological parameters that mostly affect tissue ingrowth and mechanical behavior. For instance, porosity and weight affect the intensity of foreign‐body reaction and the chance of adhesion formation between meshes and abdominal organs [10, 11, 12], while pores shape and dimensions appear more influent than the overall mesh porosity [13]. With regard to mechanical parameters, many test methods have been developed to assess the mechanical characteristic of these textile materials during the last decades. Some of them followed in vitro standard test methods (e.g., uniaxial tensile test, biaxial tensile test), while others tried to reproduce in vivo solicitation patterns (e.g., ball burst test, suture retention test) [14, 15, 16]. However, the lack of consistency among all the test set‐ups affects literature findings, preventing performance comparisons between different devices [17, 18, 19].

Although the crucial mechanical parameters for ensuring patient comfort are stiffness and compliance of the meshes indeed [20, 21], both morphological parameters (such as porosity, thickness, pore dimensions, shape, and orientation) and mechanical properties (including mesh elasticity, isotropy, and strength) actually play a role in the in vivo incorporation of the mesh and patient comfort after implantation [22, 23]. Nevertheless, the correlation between these critical aspects has only been analyzed theoretically [24]. Still, quantifying the relationship between morphological and mechanical parameters could lead to a greater understanding and awareness in mesh designs [25].

A comprehensive understanding of how the knitted pattern's structure influences implant performance would benefit all stakeholders in hernia surgery, including surgeons, patients, as well as manufacturers. At present, to commercialize hernia meshes, manufacturers must perform various mechanical and biochemical tests in order to demonstrate compliance with requirements. Moreover, if the designed morphology of a new mesh differs from already accepted ones, clinical trials are mandatory before it can be sold in the global market. This process results in high certification costs for these implantable devices, consequently leading to higher product prices in the market. In 2017, over 20 million hernias were surgically treated, and a recent market research estimates 12 billion of revenue for the global market by 2028 [1]. In light of this, the possibility to predict mechanical properties based on morphological parameters could potentially reduce the number of tests needed to characterize hernia meshes and, as a consequence, the certification costs of these devices.

The primary aim of this study is therefore to determine how the mechanical properties of various monofilament PP warp‐knitted meshes are influenced by their structural textile characteristics and which morphological properties are more relevant for each mechanical parameter. Additionally, this research seeks to formulate predictive models able to estimate mechanical parameters based on readily accessible morphological properties. Images acquired on nine hernia meshes in a previous study are used to extract meaningful morphological parameters representative of pore geometry and orientation [26]. These parameters are then correlated to twelve mechanical properties collected through a previously developed testing protocol [27].

## 2 Materials and Methods

### 2.1 Mechanical Parameters

The mechanical parameters were derived exclusively from lightweight (LW) and standard weight (SW) meshes [28]. A total of six LW meshes and three SW meshes from four different manufacturers were included in the testing. The adopted testing protocol, better detailed in [27], is briefly described in the following.

The protocol consists of three experimental set‐ups designed to conduct: uniaxial tensile test, ball burst test, and suture retention test. All the tests were conducted at a room temperature of 25°C, under displacement‐controlled conditions by means of a universal testing machine, Instron E3000 (INSTRON, Norwood, MA, USA). The specimens used in the uniaxial tensile test had a dogbone shape with a gauge length of 20 mm and were subjected to traction at a rate of 20 mm/min. To acquire the specimens elongation during uniaxial tensile tests, a DIC system, specifically the VIC‐3D system (Isi‐sys GmbH, Kassel, Germany), was utilized to track markers previously manually sewn onto the threads of the meshes. Data were post‐processed in Matlab (version 9.14.0 (R2023a). Natick, Massachusetts: The MathWorks Inc.). Two sampling directions, “weak” and “strong,” were considered in uniaxial tensile tests and suture retention tests for PP meshes. The “strong” direction was identified by comparing the failure force of the specimens between the two directions. Circular specimens with a 55 mm diameter were utilized in the ball burst test and penetrated with a steel sphere (diameter equal to 20 mm) at a rate of 300 mm/min as suggested by ASTM D6797‐15. For the suture retention test, 70 × 55 mm rectangular specimens were clamped at the upper pneumatic grip of the testing machine and moved vertically at a rate of 300 mm/min after inserting an Assusteel wire 10 mm from the lower edge. For each test, characteristic parameters were extracted related to maximum force, tension, strain, and stiffness. All the computed mechanical parameters are described in Table 1.

**TABLE 1:** Mechanical parameters computed for all the nine tested hernia meshes from the data of the three test set‐ups.

Mechanical test	Parameters and definition	Abbreviations
Ball burst test	Bursting force *Maximum force recorded during the test*	BF [N]
	Maximum membrane tension *Membrane tension in correspondence of BF*	MTmax [N/cm]
	Maximum dilatational strain *Dilatational strain in correspondence of MTmax*	DSmax [%]
	Dilatational strain at 16 N/cm *Dilatational strain in correspondence of 16 N/cm of membrane tension*	DS16 [%]
Uniaxial tensile test	Uniaxial tension at rupture—weak direction *Uniaxial tension recorded before rupture in the weak direction*	UTR—weak [N/cm]
	Uniaxial tension at rupture—strong direction *Uniaxial tension recorded before rupture in the strong direction*	UTR—strong [N/cm]
	Strain at rupture—weak direction *Strain of the specimen in correspondence of UTR—weak*	SR—weak [%]
	Strain at Rupture—strong direction *Strain of the specimen in correspondence of UTR—strong*	SR—strong [%]
	Secant stiffness—weak direction *Slope of the secant line passing through 10% of deformation in the weak direction*	k—weak [N/mm]
	Secant stiffness—strong direction *Slope of the secant line passing through 10% of deformation in the strong direction*	k—strong [N/mm]
Suture retention test	Suture Retention Strength—weak direction *Force value computed as suggested in ASTM D2261‐13 standard for the weak direction*	SRS—weak [N]
	Suture Retention Strength—strong direction *Force value computed as suggested in ASTM D2261‐13 standard for the strong direction*	SRS—strong [N]

*Note:* The numerical values of all the computed mechanical parameters for the nine meshes are reported in Table S1 as mean ± SD.

### 2.2 Morphological Parameters

The weight and the thickness of the meshes were measured by means of a high‐precision scale (Kern PCD 300‐3, Digital electronic scale, KERN & SOHN, Ebingen, Albstadt, Germany) and a thickness gauge (ABSOLUTE Digimatic Thickness Gauge, series 547, Mitutoyo, Sakado, Japan), respectively. In order to compute other morphological parameters related to pore dimensions, orientation, and shape, high‐quality images were acquired using a CANON EOS 5D Mark II digital camera equipped with a macro photography autofocus lens (Canon EF 100 mm f/2.8 Macro USM) and a custom photographic set‐up developed in a previous study [26]. During the acquisition process, attention was paid in order to align the weak and the strong directions of the meshes to the edge of the mesh support. Subsequently, the textile and effective porosity (EP) were computed using the *poreScanner* app [29].

**FIGURE 2:**
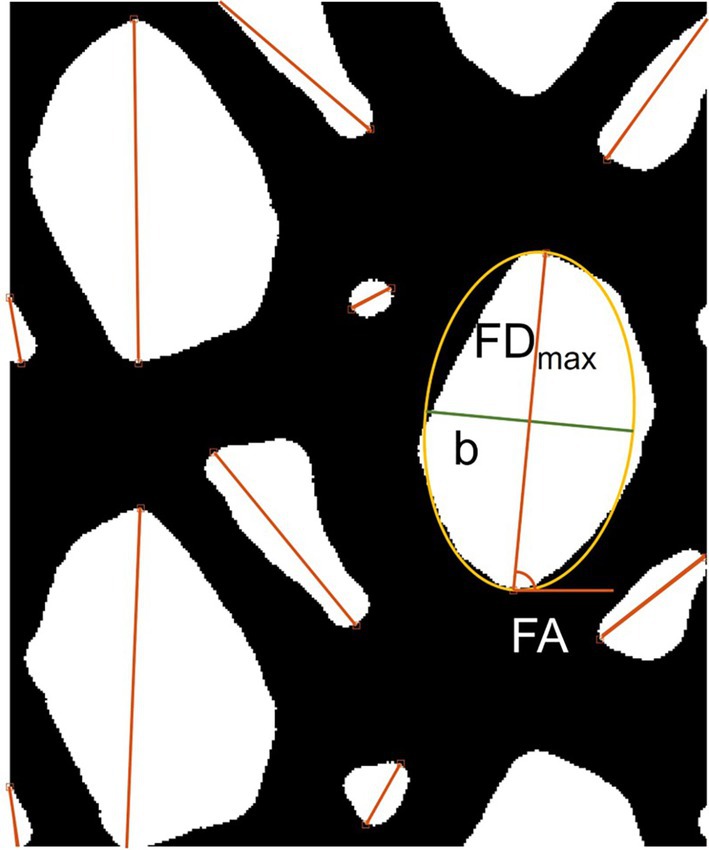
Binarized image of a SW mesh where the geometric parameters computed through regionprops function are superimposed. FD_max_: maximum Feret diameter, *b*: perpendicular axis of yellow ellipse, FA: angle of the maximum Feret diameter.

Other parameters regarding pores orientation and shape were extracted from the binarized images, starting from three parameters computed using *regionprops* function in Matlab. In detail, the pores areas, maximum Feret diameters (FD_max_) and the angles of the maximum Feret diameter (FA) were extracted for all the acquired meshes images (Figure 2). In order to achieve unique parameters that identified a predominant pores orientation and a predominant pores shape for the different meshes, a weighing factor was computed in order to take into account the relative area of each individual pore (${\text{Area}}_{i}$) compared to the total area occupied by all the pores in the mesh (${\text{Area}}_{\mathrm{tot}}$). The predominant pores orientation was thus identified through two parameters, ${\mathrm{FA}}_{w}$ defined as:

(1) $${\mathrm{FA}}_{w}=\sum_{i=1}^{n}\frac{{\text{Area}}_{i}}{{\text{Area}}_{\mathrm{tot}}}\cdot {\mathrm{FA}}_{i}$$

where ${\mathrm{FA}}_{w}$ is the weighted FA, and $\text{tanF}A_{w}$, the tangent of the weighted FA.

Moreover, assuming the overall shape of the pore as an ellipse with FD_max_ representing its longest axis, the approximate pore shape (PS) was assessed by calculating the ratio between FD_max_ and the perpendicular axis of this hypothetical ellipse (b). The predominant PS was then computed according to Equations (2) and (3):

(2) $$PS_{w}=\sum_{i=1}^{n}\frac{{\text{Area}}_{i}}{{\text{Area}}_{\mathrm{tot}}}\cdot \frac{{{\mathrm{FD}}_{\max}}_{i}}{b}$$

where

(3) $$b=2\left(\frac{{\text{Area}}_{i}}{\pi \cdot \frac{{\mathrm{FD}}_{\max_{i}}}{2}}\right)$$

The underlying concept is that when the pore approaches an almost circular shape ($PS_{w}\sim 1$), the material absence is uniformly distributed in all directions. This could result in a higher degree of isotropic behavior for the mesh [30].

A summary of the extracted morphological parameters is outlined in Table 2.

**TABLE 2:** Morphological properties computed for all the nine hernia meshes.

Morphological parameter	Abbreviations
Textile porosity	TP [%]
Effective porosity	EP [%]
Weight	W [g/m^2^]
Thickness	T [mm]
Weighted Feret angle *Sum of the angles of the maximum Feret diameter weighted on the pores Area*	FA_w_ [°]
Feret angle tangent *Tangent of FA*_*w*_	tanFA_w_
Pore shape *Sum of the ratios between maximum Feret diameter and the perpendicular diagonal of the corresponding ellipse area, weighted on the pores Area*	PS_w_

*Note:* The numerical values of all the extracted morphological parameters for the nine meshes are reported in Table S2.

### 2.3 Multivariate Linear Regression Models

#### 2.3.1 Models Creation and Selection

**FIGURE 3:**
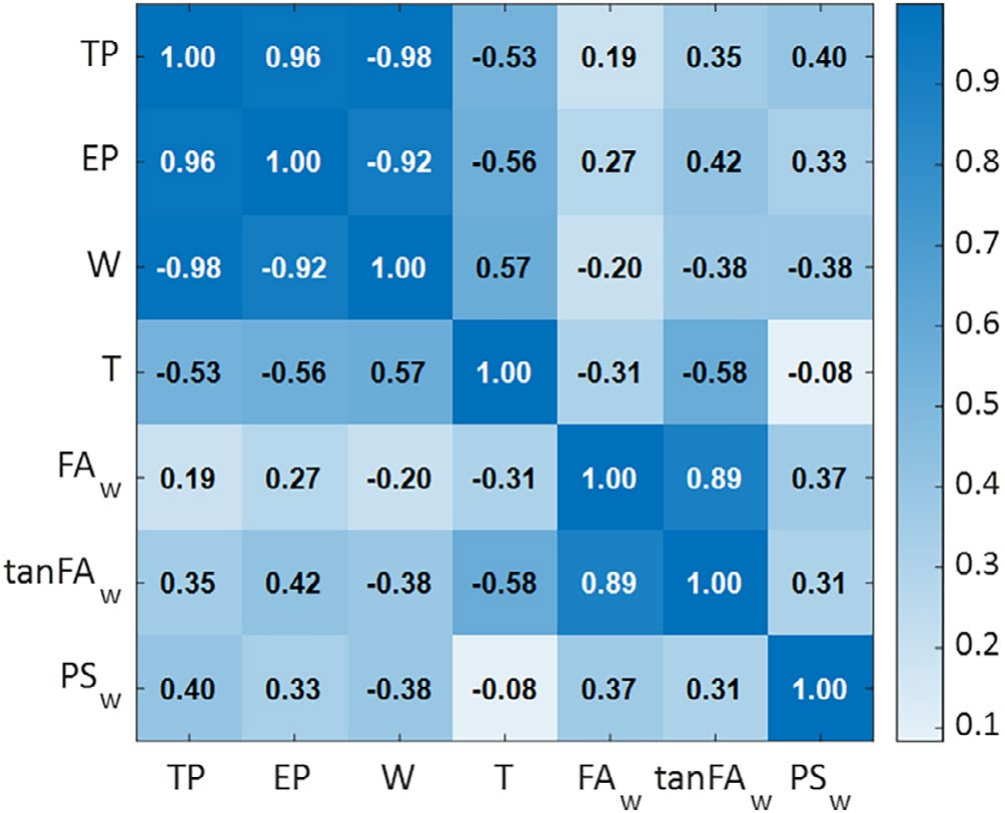
Heatmap of Pearson correlation coefficients between morphological parameters. Darker color shades indicate higher cross‐correlations, and lighter shades indicate lower correlations. Cell values represent the signed Pearson correlation coefficients.

As a preliminary step, Pearson correlation coefficients were calculated between all pairs of candidate predictors (Figure 3). Some high bivariate correlations were observed, particularly between variables with expected structural or physical relationships (e.g., textile and EP, porosity and weight). These correlations highlighted the potential for multicollinearity if too many predictors were included simultaneously. However, they were not used as exclusion criteria, since high pairwise correlation does not necessarily imply redundancy in a multivariable context: variables may still convey distinct and complementary information. Moreover, if the information provided by variables is redundant rather than complementary due to strong correlation, such variables are excluded during model selection based on multicollinearity assessment using the variance inflation factor (VIF), as detailed later in the Section 2.

Based on these considerations, a maximum of five predictors was allowed in each model. This constraint aimed to reduce multicollinearity and improve model interpretability, while preserving the ability to capture relevant structural and functional variation. To systematically explore all possible predictor combinations, 119 multivariate linear regression models were generated for each mechanical parameter (dependent variable), corresponding to all subsets of one to five predictors out of the seven available using Matlab (version 9.14.0 (R2023a). Natick, Massachusetts: The MathWorks Inc.). Multicollinearity was further assessed using the VIF, and only models in which all included variables had VIF < 8 were retained for subsequent analysis [31, 32, 33]. Subsequently, the models were evaluated for high statistical significance by applying a threshold *p‐*value less than 0.01, and ranked based on the coefficient of determination, *R* ^2^. Higher *R* ^2^ values reflect the ability of the model to explain the variance in the dependent variable and, consequently, to better predict the dependent variable itself.

#### 2.3.2 Models Validation—Leave‐One‐Out Method

Finally, for all the selected models, a leave‐one‐out (LOO) validation algorithm was carried out in order to identify overfitting, estimate model performance and robustness, and assess the predictive accuracy. Given the total number of meshes used in the model creation *n*, each multivariate regression model considered was built *n* times, each time not including the *i*th mesh in the model definition. The mechanical parameters of the *i*th mesh were then predicted using the identified multivariate regression model. The predicted values were compared to the measured ones, and to evaluate the outcome predictions, *R* ^2^ coefficients were computed through a linear regression, comparing the data to the 45‐degree line, which represents a perfect overlap.

**FIGURE 4:**
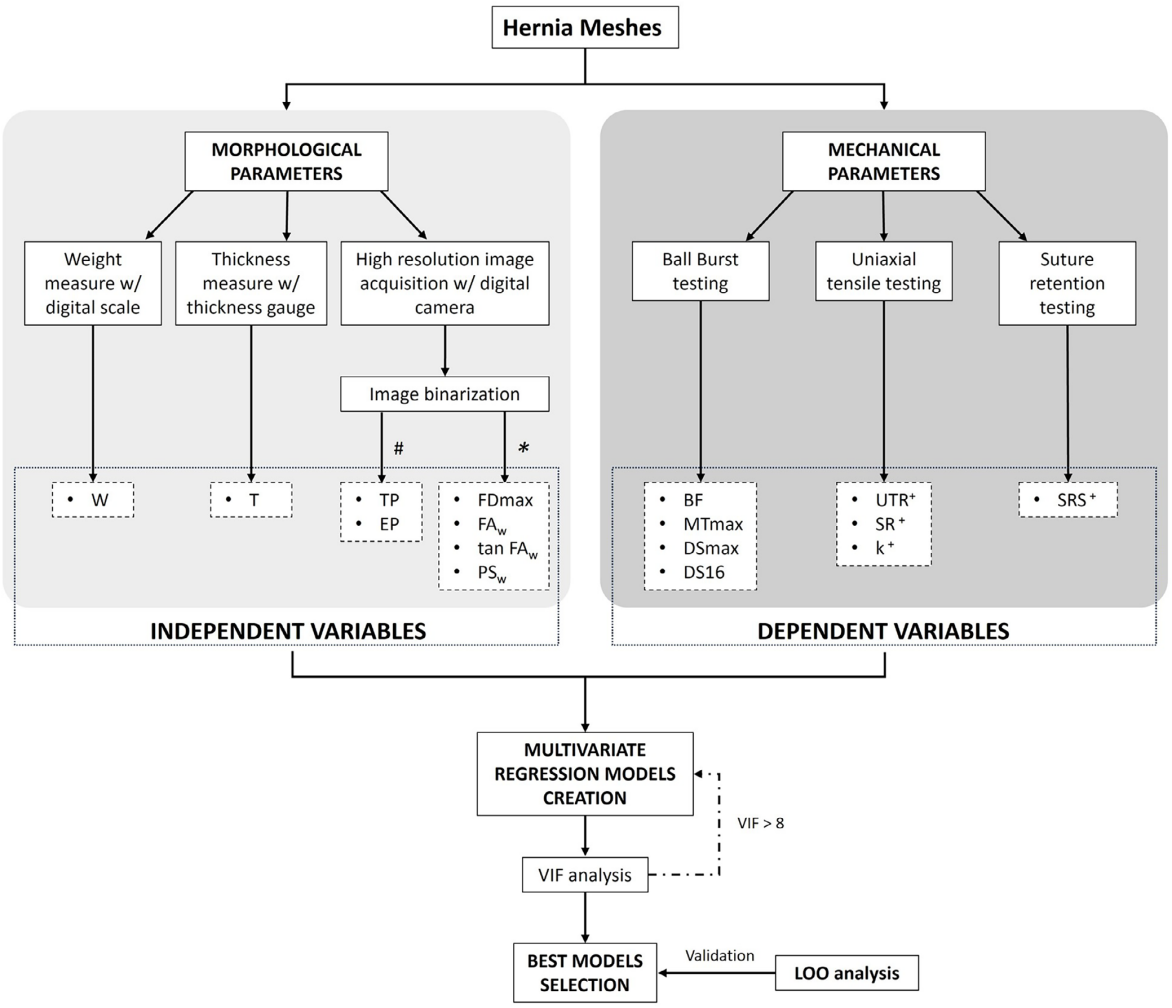
Schematic overview of the workflow. Various subsets of morphological parameters were adopted as independent variables to create regression models for each mechanical parameter (dependent variables) useful for the mechanical characterization of hernia meshes. The significance of the models was assessed by imposing a threshold on the *p* value. The multicollinearity of the independent variables was checked through a VIF analysis, and all the subsets with a VIF < 8 were considered for the best model selection based on an *R* ^2^ ranking. Finally, LOO analyses were conducted for the validation of each model. In the scheme, the dashed boxes refer to the variables used in the regression models. #: Computed through the poreScanner app [29]; *: computed through the Matlab function regionprops; +: two tested directions.

A scheme of the overall workflow for the regression models implementation is reported in Figure 4.

Eventually, the extraction of the morphological parameters related to pore shape and orientation, as well as the mechanical parameters predicted through the multivariate regression models presented here, was implemented in the *poreScanner 2.0* app [34]. In order to activate the prediction section in the new version of the app, the user needs to input high‐resolution images of the mesh along with the weight, thickness, and an identification name. In addition, the stronger direction between the two principal directions of the knitted pattern is required to reorient the mesh for the subsequent evaluation of mechanical parameters.

## 3 Results

A total of 1428 regression models were created. Among these, 171 met the imposed *p* value threshold and were therefore checked for collinearity and ranked in accordance with *R* ^2^. See Appendix A for further insight into the number of acceptable models for each mechanical parameter.

### 3.1 Models Selection

The model selected for each of the 12 models consists of a distinct subset of variables. Table 3 highlights the variables included in each model, along with their respective weights. Each model is indeed the result of a linear combination of the weights and the independent variables, with an added intercept term. The obtained coefficient of determination and *p* value are indicated.

**TABLE 3:** Weights for each selected independent variable (on columns) according to each of the 12 models (on rows).

	Intercept	TP	EP	W	T	FA_w_	tanFA_w_	PS_w_	*R* ^2^	*p*
BF	1037.68		−8.72	−3.18			2.37	−76.79	**0.97**	**0.004**
MTmax	246.07		−1.41	−0.60	−110.25	−0.42			**0.94**	**0.010**
DSmax	−0.33		−0.47		135.13	0.56		−14.38	**0.95**	**0.009**
DS16	−35.98		0.17	0.08	73.32		2.49		**0.82**	0.089*
UTR—weak	122.04		−0.52		−141.25		−4.10	1.79	**0.95**	**0.007**
UTR—strong	76.90		−1.01	−0.21	35.10				**0.92**	**0.004**
SR—weak	144.92		−0.66				−4.70	−22.02	**0.71**	0.084*
SR—strong	42.74			0.44					**0.59**	0.016*
k—weak	−0.67		0.05	0.06	−8.65		−0.44	0.78	**0.95**	0.034*
k—strong	4.95		−0.02			−0.03			**0.78**	**0.010**
SRS—weak	93.02	−1.18			23.11		0.92		**0.90**	**0.007**
SRS—strong	116.37		−0.53		−78.62		−2.16	−5.31	**0.97**	**0.003**

*Note:* *R* ^2^ and the *p* value are also reported in bold. *p value greater than the imposed threshold of 0.01.

Table 3 pointed out high values of *R* ^2^ for all the models. The most recurrent variables in the models are EP, thickness (T) and tanFA_w_, selected in nine out of 12 models. Additionally, in all the models except for SR‐strong, a porosity metric and a pore orientation or shape parameter were always included. For two models (marked with an asterisk in Table 3), it was not possible to achieve a model that met the imposed threshold on the *p* value, so a model with a higher *p* value is selected. Lastly, for k‐weak, a model with a higher *p* value was chosen because neither of the two models with a *p* value < 0.01 met the threshold for multicollinearity.

For the selected models, other metrics were computed to compare the 12 models, to evaluate the models performance, and to assess the error (Table 4).

**TABLE 4:** Regression performance metrics for the 12 models, including the coefficient of determination (*R* ^2^), the adjusted *R* ^2^ (*R* ^2^_adj_), and the relative standard error (RSE).

	*R* ^2^	*R* ^2^_adj_	RSE
BF	0.97	0.87	0.03
MTmax	0.94	0.88	0.06
DSmax	0.95	0.81	0.05
DS16	0.82	0.62	0.18
UTR—weak	0.95	0.84	0.05
UTR—strong	0.92	0.63	0.08
SR—weak	0.71	0.29	0.29
SR—strong	0.59	0.53	0.41
k—weak	0.95	0.77	0.05
k—strong	0.78	0.71	0.22
SRS—weak	0.90	0.72	0.10
SRS—strong	0.97	0.75	0.03

Similar values were obtained overall by comparing *R* ^2^ and *R* ^2^_adj_ with the exception of model SR—weak, in which the drop in *R* ^2^_adj_ suggests potential overfitting. This may be due to the limited contribution of the selected predictors in SR–weak, despite the good nominal *R* ^2^. Eight models show RSE ≤ 0.10, indicating that the relative error in these models is below 10% of the observed mean values, which is generally considered a threshold for high model accuracy.

### 3.2 Models Validation

The LOO analyses conducted on the twelve selected models revealed good predictive capabilities in five models (*R* ^2^ > 0.65) and moderate ones in one model (*R* ^2^ > 0.55). As expected, the remaining models were those with the worst *p* values and low RSE.

**FIGURE 5:**
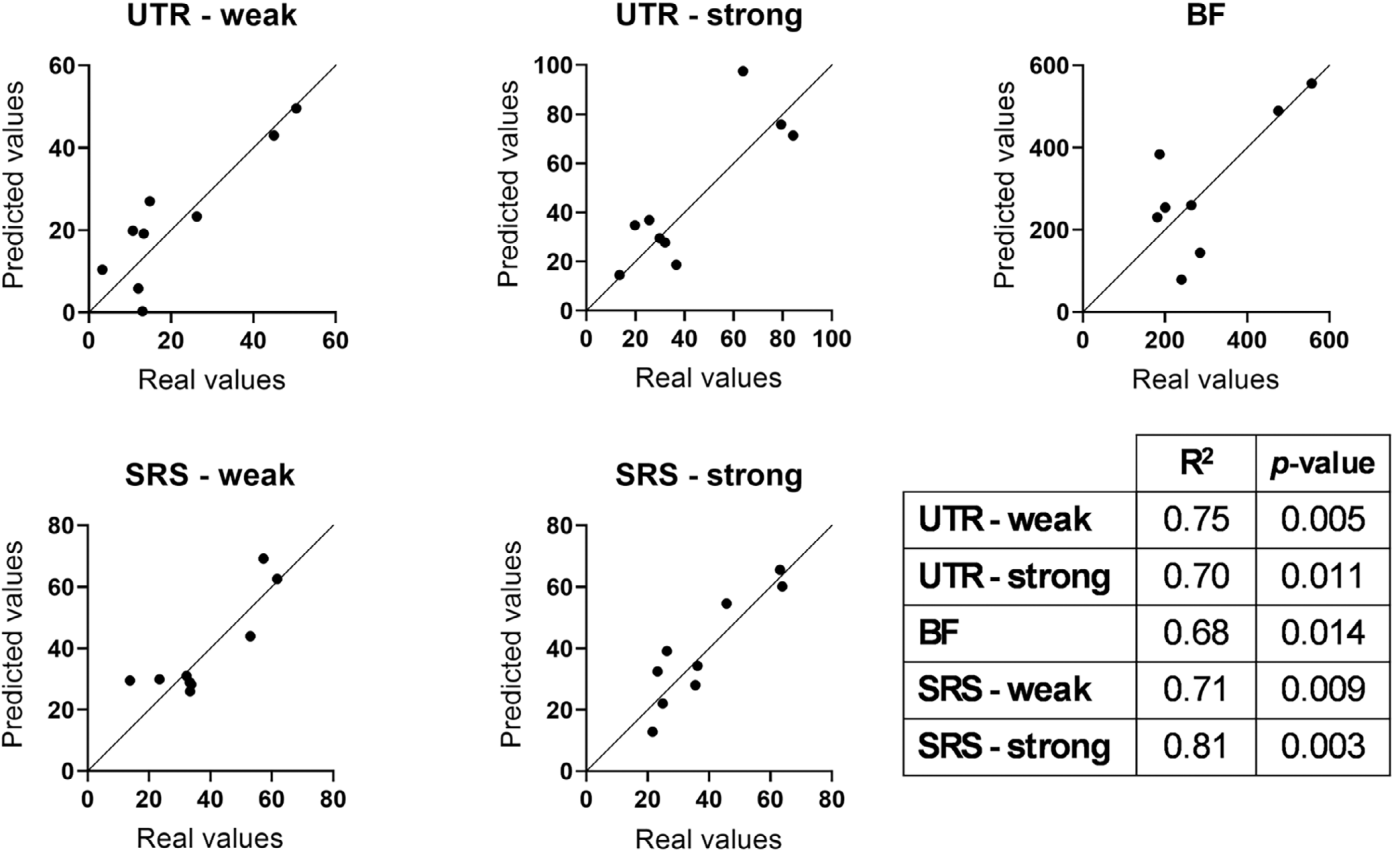
Graphs representing the predicted versus the measured values for the best models, together with the 45‐degree line. The table in the bottom right corner of the figure reports the coefficient of determination and the *p* value for each parameter.

Figure 5 depicts the predicted values versus the measured values obtained in the LOO for the best models together with the first and third quadrant bisector.

## 4 Discussion

Morphological characteristics of hernia meshes are typically associated with in vivo integration and wound healing [23, 35]. However, the successful host integration and patient comfort after implantation also rely on the mechanical characteristics of the implant. Nevertheless, how the microstructure of the meshes affects their mechanical behavior remains unclear [36]. In this context, the possibility of obtaining information about the mechanical behavior of a synthetic surgical mesh based on its morphological parameters could potentially contribute to the reduction of recurrence and failures that persist in abdominal repair surgery, enhancing clinical outcomes. This study thus aimed to assess the link between these two aspects through multivariate correlation analyses. In this perspective, to quantify morphological characteristics able to distinguish between different meshes, seven morphological parameters were computed for nine meshes, addressing mesh weight, thickness, and two porosity metrics, as well as pore shape and orientation. While the impact of the fabric structure on the mechanical behavior of meshes has been partly explored through comparisons of various warp‐knitted patterns, a comprehensive quantification of the morphological properties of the fabrics that might be considered and modified in the design of the device in order to obtain the necessary mechanical properties for specific applications has not yet been assessed [37]. The additional morphological parameters computed in this study, and the subsequent development of multivariate regression models were therefore undertaken in order to fill this gap, allowing for the determination of which and how morphological parameters would mostly affect the mechanical behavior.

The selection of the models was based on a threshold *p* value and a ranking based on the determination coefficient. The three dependent variables that do not adhere to the threshold are strain‐related variables. Among them, the worst performance was obtained by DS16, a parameter deeply affected by computational method. This likely decreases the correlation between this parameter and the morphology of the meshes. Overall, the performed LOO analysis confirmed a strong relationship between the predictivity of the model and high *p* value, as models with a *p* value higher than 0.01 consistently exhibited the worse performance. The tangent of the weighted Feret angle resulted in the most relevant morphological parameter related to pore orientation (i.e., FA_w_ and tanFA_w_). Out of the 10 models that selected a pore orientation parameter, only three utilized FA_w_. Interestingly, although nowadays surgical meshes are classified according to weight, weight was the least prioritized among the morphological parameters, showing limited correlation with mechanical behavior. In fact, the negligible role played by meshes weight on biocompatibility had already been previously identified, especially if compared to pore size and the overall textile microstructure [13, 38]. In this regard, the better surgical outcomes in terms of pain and shorter recovery time, often associated with the use of LW mesh, might indeed be related to the tendency to have larger pore sizes in LW meshes in comparison to the heavyweight ones [22]. The crucial role of pore dimension and therefore porosity is indeed confirmed by our models. One of the two porosity parameters considered (i.e., textile or EP) was selected in 11 models, confirming its pivotal role [39]. Moreover, given the established significance of pore shape found in literature [40, 41, 42] in host integration and in many mechanical parameters (e.g., anisotropy, stiffness, dilatational strain), the PS_w_ parameter, indicating pore roundness, was computed and considered in the creation of the regression models. Conversely, parameters related to yarn diameter and number of threads along the warp and weft directions were not considered pivotal parameters in this study. Indeed, since all light meshes have a diameter of 120 μm and all standard meshes have a diameter of 180 μm, yarn diameter was not considered an informative morphological parameter. On the other hand, the difficulties in accurately assessing the number of threads, especially in standard meshes with intricate patterns, were deemed not worthwhile. However, this omission was considered acceptable since the correlation between the number of threads and other morphological parameters, such as porosity and thickness of knitted devices, has already been established in the literature [43, 44, 45, 46].

The outlined study presents some limitations which will be mentioned in the following. First, morphological parameters were extracted as single representative values per mesh, and intra‐mesh variability was not assessed. While this approach was appropriate for their use as inputs in the regression models, it prevented the evaluation of local morphological heterogeneity, which may affect mesh performance. Additionally, the dataset used for developing the multivariate regression models was relatively small, reflecting the challenges associated with acquiring a representative and diverse set of commercial mesh samples. Small datasets may not capture the full diversity of the population, posing difficulties in model generalization. Nevertheless, aiming to ensure representative data collection, different meshes were selected from the weight categories of the most implanted devices (LW and SW). Furthermore, the limited size of the dataset precluded the use of any validation methods other than LOO [47, 48]. Although data augmentation through synthetic datasets could potentially improve model robustness, this approach requires validated computational models capable of accurately reproducing meshes mechanical behavior, which are currently under development and were not available for this study. LOO, however, can be less reliable with small datasets since leaving out a single data point for validation may result in a training set that lacks sufficient representativeness. The repeated training on a reduced dataset might also exacerbate this issue in LOO, potentially leading to an underestimation of the assessed predictive capabilities. Despite these drawbacks, the results derived from the LOO analysis pointed out robust predictive capabilities for several models. This highlights the potential utility of these models in predicting the mechanical performance of different meshes based on morphological parameters extracted from images. Interestingly, the models exhibiting the highest performance (*R* ^2^ > 0.65, see Figure 4) included parameters that manufacturers are required to provide for the certification of these devices. This corroborates the applicability of the proposed predictive models to infer the mechanical performance of a mesh from its microstructural features as well as the automatic extraction of additional morphological parameters implemented in the last version of the *poreScanner* app [34]. Moreover, it is worth noting that both weak and strong directions, corresponding to sampling orientations relative to the mesh weave, led to accurate models in several cases (e.g., UTR and SRS). This suggests that the selected morphological descriptors retain predictive relevance regardless of specimen orientation, indicating a certain robustness despite the known anisotropy of the material. Although no universally accepted thresholds exist for interpreting *R* ^2^ values, common guidelines from Partial Least Squares Structural Equation Modeling (PLS‐SEM) suggest that values of 0.75, 0.50, and 0.25 can be considered substantial, moderate, and weak, respectively [49, 50]. It is well recognized that appropriate thresholds may vary depending on the scientific domain, data complexity, and inherent variability [51, 52]. In this context, the chosen threshold of *R* ^2^ > 0.65 to denote “good” predictive ability reflects a value intermediate between the moderate and substantial benchmarks. Similarly, models with *R* ^2^ > 0.55 were classified as having moderate predictive power. Furthermore, the possibility to validate the models with other commercially available or specifically designed meshes will significantly enhance the comprehension of the interplay between mechanical and morphological parameters. While the present study focused on interpretable regression models based on physically meaningful morphological features, primarily due to the limited size of the dataset, future research could investigate machine learning approaches to capture more complex, non‐linear relationships. The generation and use of synthetic datasets, combined with validation on completely unseen meshes, may become feasible as larger datasets become available, potentially enhancing model robustness and generalizability. Such advances would support more data‐driven strategies for the design and selection of surgical meshes. In particular, using these models during the design phase of new meshes could enable the creation of devices with the required mechanical characteristics tailored to different types of patients while minimizing the amount of material used and optimizing pore shape to promote tissue ingrowth, thus avoiding infection or unwanted biochemical responses. Additionally, our findings could be useful during surgical decision‐making in order to aid medical teams in the selection of the best surgical mesh, considering the anatomical region of implantation and patient variability.

## 5 Conclusions

In conclusion, this comprehensive analysis sheds light on the strong correlation between morphological and mechanical characteristics of hernia meshes. The consistent selection of thickness, porosity metrics, and pore orientation‐related variables underscores their critical roles in the mechanical behavior of hernia meshes. The developed models were implemented in the new version of the free‐to‐use app, *poreScanner 2.0*. The updated version aims to encourage its adoption, making these insights accessible for a wider audience in this field.

## Ethics Statement

The authors have nothing to report.

## Conflicts of Interest

The authors declare no conflicts of interest.

## Supporting information


**Table S1:** Mechanical parameters for the nine meshes in the three test setups.
**Table S2:** Morphological parameters for the nine meshes.

## Data Availability

The data that support the findings of this study are available on request from the corresponding author. The data are not publicly available due to privacy or ethical restrictions.

## Appendix A

Table A1 reports the number of models that met the imposed *p* value and subsequently the VIF threshold.

**TABLE A1:** Number of models meeting the imposed thresholds. First column refers to the number of models out of the 119 created for each mechanical parameter that satisfies the imposed *p* value; second column refers to the number of models that additionally fulfill the VIF threshold.

		Number of models	
		*p* < 0.01	VIF ≤ 8
Mechanical parameters	BF	35	19
	MTmax	8	1
	DSmax	3	3
	DS16	0	0
	UTR—weak	7	5
	UTR—strong	37	22
	SR—weak	0	0
	SR—strong	0	0
	k—weak	1	0
	k—strong	1	1
	SRS—weak	33	18
	SRS—strong	46	24

## References

[1] K. Baylón , P. Rodríguez‐Camarillo , A. Elías‐Zùñiga , J. A. Díaz‐Elizondo , R. Gilkerson , and K. Lozano , “Past, Present and Future of Surgical Meshes: A Review,” Membranes (Basel) 7, no. 47 (2017): 1–23. 10.3390/membranes7030047 PMC5618132 28829367

[2] Y. Bilsel and I. Abci , “The Search for Ideal Hernia Repair; Mesh Materials and Types,” International Journal of Surgery 10, no. 6 (2012): 317–321. 22588090 10.1016/j.ijsu.2012.05.002

[3] A. Rastegarpour , M. Cheung , M. Vardhan , M. M. Ibrahim , C. E. Butler , and H. Levinson , “Surgical Mesh for Ventral Incisional Hernia Repairs: Understanding Mesh Design,” Canadian Journal of Plastic Surgery 24, no. 1 (2016): 41–50. 10.4172/plastic-surgery.1000955 PMC4806756 27054138

[4] C. R. Deeken and S. P. Lake , “Mechanical Properties of the Abdominal Wall and Biomaterials Utilized for Hernia Repair,” Journal of the Mechanical Behavior of Biomedical Materials 74 (2017): 411–427. 28692907 10.1016/j.jmbbm.2017.05.008

[5] K. Junge , U. Klinge , A. Prescher , P. Giboni , M. Niewiera , and V. Schumpelick , “Elasticity of the Anterior Abdominal Wall and Impact for Reparation of Incisional Hernias Using Mesh Implants,” Hernia 5, no. 3 (2001): 113–118. 11759794 10.1007/s100290100019

[6] Y. Ozog , J. Deprest , K. Haest , F. Claus , D. De Ridder , and E. Mazza , “Calculation of Membrane Tension in Selected Sections of the Pelvic Floor,” International Urogynecology Journal 25, no. 4 (2014): 499–506. 24146072 10.1007/s00192-013-2253-1

[7] C. Song , A. Alijani , T. Frank , G. B. Hanna , and A. Cuschieri , “Mechanical Properties of the Human Abdominal Wall Measured in Vivo During Insufflation for Laparoscopic Surgery,” Surgical Endoscopy and Other Interventional Techniques 20, no. 6 (2006): 987–990. 16738998 10.1007/s00464-005-0676-6

[8] F. Köckerling , “Recurrent Incisional Hernia Repair—An Overview,” Frontiers in Surgery 6 (2019): 26. 31139632 10.3389/fsurg.2019.00026 PMC6527885

[9] U. Klinge , B. Klosterhalfen , M. Müller , and V. Schumpelick , “Foreign Body Reaction to Meshes Used for the Repair of Abdominal Wall Hernias,” European Journal of Surgery 165, no. 7 (1999): 665–673. 10.1080/11024159950189726 10452261

[10] J. Conze , R. Rosch , U. Klinge , et al., “Polypropylene in the Intra‐Abdominal Position: Influence of Pore Size and Surface Area,” Hernia 8 (2004): 365–372. 15309687 10.1007/s10029-004-0268-8

[11] K. Junge , U. Klinge , R. Rosch , B. Klosterhalfen , and V. Schumpelick , “Functional and Morphologic Properties of a Modified Mesh for Inguinal Hernia Repair,” World Journal of Surgery 26 (2002): 1472–1480. 12297937 10.1007/s00268-002-6444-z

[12] D. A. Raptis , B. Vichova , J. Breza , J. Skipworth , and S. Barker , “A Comparison of Woven Versus Nonwoven Polypropylene (PP) and Expanded Versus Condensed Polytetrafluoroethylene (PTFE) on Their Intraperitoneal Incorporation and Adhesion Formation,” Journal of Surgical Research 169, no. 1 (2011): 1–6. 20400113 10.1016/j.jss.2009.12.014

[13] S. P. Lake , S. Ray , A. M. Zihni , D. M. Thompson , J. Gluckstein , and C. R. Deeken , “Pore Size and Pore Shape – But Not Mesh Density – Alter the Mechanical Strength of Tissue Ingrowth and Host Tissue Response to Synthetic Mesh Materials in a Porcine Model of Ventral Hernia Repair,” Journal of the Mechanical Behavior of Biomedical Materials 42 (2015): 186–197. 25486631 10.1016/j.jmbbm.2014.11.011

[14] A. Cordero , B. Hernández‐gascón , G. Pascual , J. M. Bellòn , B. Calvo , and E. Pena , “Biaxial Mechanical Evaluation of Absorbable and Nonabsorbable Synthetic Surgical Meshes Used for Hernia Repair: Physiological Loads Modify Anisotropy Response,” Annals of Biomedical Engineering 44 (2015): 2181–2188. 26620778 10.1007/s10439-015-1503-4

[15] B. J. Eliason , M. M. Frisella , B. D. Matthews , and C. R. Deeken , “Effect of Repetitive Loading on the Mechanical Properties of Synthetic Hernia Repair Materials,” Journal of the American College of Surgeons 213, no. 3 (2011): 430–435. 21705242 10.1016/j.jamcollsurg.2011.05.018

[16] S. Est , M. Roen , T. Chi , et al., “Multi‐Directional Mechanical Analysis of Synthetic Scaffolds for Hernia Repair,” Journal of the Mechanical Behavior of Biomedical Materials 71 (2017): 43–53. 28259784 10.1016/j.jmbbm.2017.02.009

[17] S. Sahoo , K. R. Delozier , A. Erdemir , and K. A. Derwin , “Clinically Relevant Mechanical Testing of Hernia Graft Constructs,” Journal of the Mechanical Behavior of Biomedical Materials 41 (2015): 177–188. 25460414 10.1016/j.jmbbm.2014.10.011

[18] C. R. Deeken , M. S. Abdo , M. M. Frisella , and B. D. Matthews , “Physicomechanical Evaluation of Polypropylene, Polyester, and Polytetrafluoroethylene Meshes for Inguinal Hernia Repair,” Journal of the American College of Surgeons 212, no. 1 (2011): 68–79. 21115372 10.1016/j.jamcollsurg.2010.09.012

[19] S. Todros , P. Pachera , P. G. Pavan , and A. N. Natali , “Investigation of the Mechanical Behavior of Polyester Meshes for Abdominal Surgery: A Preliminary Study,” Journal of Medical and Biological Engineering 38, no. 4 (2018): 654–665.

[20] S. Kalaba , E. Gerhard , J. S. Winder , E. M. Pauli , R. S. Haluck , and J. Yang , “Design Strategies and Applications of Biomaterials and Devices for Hernia Repair,” Bioactive Materials 1, no. 1 (2016): 2–17. 28349130 10.1016/j.bioactmat.2016.05.002 PMC5365083

[21] M. A. Konerding , M. Bohn , T. Wolloscheck , et al., “Maximum Forces Acting on the Abdominal Wall: Experimental Validation of a Theoretical Modeling in a Human Cadaver Study,” Medical Engineering & Physics 33, no. 6 (2011): 789–792. 21333582 10.1016/j.medengphy.2011.01.010

[22] L.‐M. Zhu , “Mesh Implants: An Overview of Crucial Mesh Parameters,” World Journal of Gastrointestinal Surgery 7, no. 10 (2015): 226–236. 26523210 10.4240/wjgs.v7.i10.226 PMC4621472

[23] C. W. See , T. Kim , and D. Zhu , “Hernia Mesh and Hernia Repair: A Review,” Engineered Regeneration 1 (2020): 19–33.

[24] L. Miao , F. Wang , L. Wang , T. Zou , G. Brochu , and R. Guidoin , “Physical Characteristics of Medical Textile Prostheses Designed for Hernia Repair: A Comprehensive Analysis of Select Commercial Devices,” Materials (Basel) 8, no. 12 (2015): 8148–8168. 28793704 10.3390/ma8125453 PMC5458830

[25] S. Todros , P. G. Pavan , P. Pachera , and A. N. Natali , “Synthetic Surgical Meshes Used in Abdominal Wall Surgery: Part II—Biomechanical Aspects,” Journal of Biomedical Materials Research, Part B: Applied Biomaterials 105, no. 4 (2017): 892–903. 26687728 10.1002/jbm.b.33584

[26] V. Giacalone , V. Civilini , A. L. Audenino , and M. Terzini , “Quantifying Mesh Textile and Effective Porosities: A Straightforward Image Analysis Procedure for Morphological Analysis of Surgical Meshes,” Computer Methods and Programs in Biomedicine 242 (2023): 107850. 37865005 10.1016/j.cmpb.2023.107850

[27] V. Civilini , V. Giacalone , A. L. Audenino , and M. Terzini , “A Reliable and Replicable Test Protocol for the Mechanical Evaluation of Synthetic Meshes,” Journal of the Mechanical Behavior of Biomedical Materials 144 (2023): 105987. 37413894 10.1016/j.jmbbm.2023.105987

[28] A. Coda , R. Lamberti , and S. Martorana , “Classification of Prosthetics Used in Hernia Repair Based on Weight and Biomaterial,” Hernia 16, no. 1 (2012): 9–20. 21837484 10.1007/s10029-011-0868-z

[29] V. Giacalone , V. Civilini , A. Audenino , and M. Terzini , PoreScanner (Zenodo, 2023).

[30] P. Liu , H. Shao , N. Chen , and J. Jiang , “Physico‐Mechanical Performance Evaluation of Large Pore Synthetic Meshes With Different Textile Structures for Hernia Repair Applications,” Fibers and Textiles in Eastern Europe 2, no. 128 (2018): 79–86.

[31] M. H. Kutner , C. J. Nachtshein , and J. Neter , Applied Linear Regression Models, 4th ed. (McGraw‐Hill/Irwing, 2004).

[32] H. Midi , S. K. Sarkar , and S. Rana , “Collinearity Diagnostics of Binary Logistic Regression Model,” Journal of Interdisciplinary Mathematics 13, no. 3 (2010): 253–267.

[33] R. M. O'Brien , “A Caution Regarding Rules of Thumb for Variance Inflation Factors,” Quality and Quantity 41, no. 5 (2007): 673–690.

[34] V. Giacalone , V. Civilini , A. L. Audenino , and M. Terzini , PoreScanner (2.0) (Zenodo, 2024).

[35] H. Greca , P. Biondo , S. Time , and A. Mansur , “The Influence of Differing Pore Sizes on the Biocompatibility of Two Polypropylene Meshes in the Repair of Abdominal Defects,” Hernia 5, no. 2 (2001): 59–64. 11505649 10.1007/s100290100001

[36] T. Liu , Z. Ye , B. Yu , W. Xuan , J. Kang , and J. Chen , “Biomechanical Behaviors and Visco‐Hyperelastic Mechanical Properties of Human Hernia Patches With Polypropylene Mesh,” Mechanics of Materials 176 (2023): 104529.

[37] M. Mirjavan , A. Asayesh , A. Asghar , and A. Jeddi , “The Effect of Fabric Structure on the Mechanical Properties of Warp Knitted Surgical Mesh for Hernia Repair,” Journal of the Mechanical Behavior of Biomedical Materials 66 (2017): 77–86. 27838593 10.1016/j.jmbbm.2016.10.016

[38] D. Weyhe , I. Schmitz , O. Belyaev , R. Grabs , W. Uhl , and V. Zumtobel , “Experimental Comparison of Monofile Light and Heavy Polypropylene Meshes: Less Weight Does Not Mean Less Biological Response,” World Journal of Surgery 30 (2006): 1586–1591. 16855805 10.1007/s00268-005-0601-0

[39] U. Klinge and B. Klosterhalfen , “Modified Classiffication of Surgical Meshes for Hernia Repair Based on the Analyses of 1,000 Explanted Meshes,” Hernia 16, no. 3 (2012): 251–258. 22562353 10.1007/s10029-012-0913-6 PMC3360857

[40] C. Brune , S. Vogt , C. Peiper , K. Brinker , and J. Trzewik , “A New Universal Pore Measurement and Clustering Approach for Surgical Meshes,” bioRxiv (2018): 1–5.

[41] K. M. Knight and P. A. Moalli , “Preventing Mesh Pore Collapse by Designing Mesh Pores With Auxetic Geometries: A Comprehensive Evaluation via Computational Modeling,” Journal of Biomechanical Engineering 140 (2018): 1–8. 10.1115/1.4039058 PMC7104754 29350744

[42] H. Shao , J. Li , N. Chen , G. Shao , J. Jiang , and Y. Yang , “Experimental Study on Bi‐Axial Mechanical Properties of Warp‐Knitted Meshes With and Without,” Materials (Basel) 11, no. 10 (2018): 1999. 30332825 10.3390/ma11101999 PMC6213491

[43] S. Benltoufa , F. Fayala , and S. Ben Nasrallah , “Porosity Determination of Jersey Structure,” Autex Research Journal 7, no. 1 (2007): 63–69.

[44] M. K. Imrith , R. Unmar , and S. Rosunee , “Investigating the Relationship Between Knitted Fabric Porosity and Light Permeability,” Indian Journal of Materials Science 2016 (2016): 1–12.

[45] T. Ogulata , “Investigation of Porosity and Air Permeability Values of Plain Knitted Fabrics,” Fibers and Textiles in Eastern Europe 18, no. 5 (2010): 71–75.

[46] M. Owais , R. Siddiqui , and D. Sun , “Porosity Prediction of Plain Weft Knitted Fabrics,” Fibers 3 (2015): 1–11.

[47] T. Hastie , R. Tibshirani , and J. Friednam , The Element of Statistical Learning:Data Mining, Inference, and Prediction, Second ed. (Springer, 2008).

[48] T. Wong , “Performance Evaluation of Classification Algorithms by k ‐Fold and Leave‐One‐Out Cross Validation,” Pattern Recognition 48, no. 9 (2015): 2839–2846.

[49] J. F. Hair , C. M. Ringle , and M. Sarstedt , “PLS‐SEM: Indeed a Silver Bullet,” Journal of Marketing Theory and Practice 19, no. 2 (2011): 139–152.

[50] J. F. Hair , G. T. M. Hult , C. M. Ringle , and M. Sarstedt , A Primer on Partial Least Squares Structural Equation Modeling (SAGE Pubblications, 2014).

[51] D. F. Hamilton , M. Ghert , and A. H. R. W. Simpson , “Interpreting Regression Models in Clinical Outcome Studies,” Bone & Joint Research 4, no. 9 (2015): 152–153. 26392591 10.1302/2046-3758.49.2000571 PMC4678365

[52] M. Sarstedt , C. M. Ringle , and J. F. Hair , “Partial Least Squares Structural Equation Modeling,” in Handbook of Market Research (Springer International Publishing, 2021), 587–632.

