# Supplementary material for: Assessing the Impact of Morphological Parameters on the Mechanical Behavior of Synthetic Meshes. A Multivariate Regression Approach
Source: Int J Numer Method Biomed Eng. 2025 Sep 12;41(9):e70092. doi: 10.1002/cnm.70092 (PMC12429015; doi:10.1002/cnm.70092)
Supplement: Supplementary file 1 — Table S1: Mechanical parameters for the nine meshes in the three test setups. Table S2: Morphological parameters for the nine meshes. [file CNM-41-e70092-s001.docx]

**Supplementary material**

*for*

Assessing the impact of morphological parameters on the mechanical behavior of synthetic meshes. A multivariate regression approach.

Vittoria Civilini^1,2^, Alessandra Aldieri^1,2^, Vincenzo Giacalone^1,2^, Alberto L. Audenino^1,2^,

Mara Terzini^1,2^

^1^ Department of Mechanical and Aerospace Engineering, Politecnico di Torino, 10129 Turin, Italy

^2^ Polito^BIO^Med Lab, Politecnico di Torino, 10129 Turin, Italy

**Corresponding author:** Vittoria Civilini, [vittoria.civilini@polito.it](mailto:vittoria.civilini@polito.it),

Corso Duca degli Abruzzi, 24 | 10129 Torino, ITALY

**Contents**

[Table S1: Mechanical parameters for the nine meshes in the three test setups. 2](#_Toc203034770)

[Table S2: Morphological parameters for the nine meshes. 2](#_Toc203034771)

The mechanical parameters are reported in Table S1 as mean ± standard deviation, while the morphological parameters are summarized in Table S2. These descriptors were treated as independent variables in the regression models, and a single representative value per mesh was extracted using a standardized image analysis pipeline. Consequently, variability across replicates was not evaluated, and the reported values serve to characterize mesh morphology as model inputs rather than as outcome measures.

# Table S1: Mechanical parameters for the nine meshes in the three test setups.

|  |  | **Ball Burst test** | | | | **Uniaxial tensile test** | | | | | | **Suture retention test** | |
| --- | --- | --- | --- | --- | --- | --- | --- | --- | --- | --- | --- | --- | --- |
|  |  | BF  [N] | MTmax [N/cm] | Dsmax [%] | DS16 [%] | UTR [N/cm] | | SR [%] | | k [N/mm] | | SRS [N] | |
|  |  |  |  |  |  | Weak | Strong | Weak | Strong | Weak | Strong | Weak | Strong |
| **LW** | Mesh ID 1 | 240.82 ± 8.71 | 57.02 ± 1.03 | 45.09 ± 2.10 | 23.65 ± 1.42 | 3.28 ± 0.57 | 32.04 ± 3.33 | 52.58 ± 5.43 | 65.78 ± 4.63 | 0.36 ± 0.10 | 1.45 ± 0.64 | 33.76 ± 2.21 | 21.56 ± 3.52 |
|  | Mesh ID 2 | 285.37 ± 9.09 | 71.72 ± 1.89 | 41.56 ± 1.48 | 15.02 ± 1.13 | 13.33 ± 2.22 | 36.61 ± 2.75 | 52.03 ± 6.20 | 94.06 ± 10.58 | 2.07 ± 0.10 | 1.70 ± 0.27 | 33.39 ± 3.11 | 35.53 ± 2.34 |
|  | Mesh ID 3 | 263.92 ± 12.63 | 67.13 ± 1.35 | 40.88 ± 1.67 | 16.55 ± 0.69 | 26.26 ± 3.32 | 29.84 ± 2.55 | 67.76 ± 7.54 | 59.86 ± 5.38 | 2.21 ± 0.24 | 2.02 ± 0.17 | 32.24 ± 3.32 | 36.23 ± 2.82 |
|  | Mesh ID 4 | 186.46 ± 38.12 | 53.59 ± 8.54 | 34.79 ± 4.54 | 24.02 ± 6.79 | 11.99 ± 3.33 | 13.48 ± 2.17 | 107.59 ± 19.98 | 44.75 ± 12.89 | 0.08 ± 0.02 | 1.45 ± 0.64 | 23.38 ± 3.31 | 26.27 ± 1.85 |
|  | Mesh ID 5 | 200.49 ± 10.82 | 52.07 ± 0.99 | 39.86 ± 3.52 | 25.18 ± 2.22 | 10.68 ± 1.49 | 19.74 ± 1.68 | 76.48 ± 13.11 | 60.39 ± 8.08 | 1.30 ± 0.18 | 3.18 ± 0.63 | 13.77 ± 2.34 | 23.20 ± 1.72 |
|  | Mesh ID 6 | 181.07 ± 9.75 | 49.87 ± 2.51 | 36.89 ± 2.58 | 24.19 ± 1.15 | 13.03 ± 4.24 | 25.59 ± 3.67 | 69.14 ± 14.18 | 57.24 ± 4.20 | 1.39 ± 0.19 | 2.36 ± 0.26 | 33.20 ± 1.25 | 24.91 ± 4.06 |
| **SW** | Mesh ID 7 | 557.11 ± 65.94 | 77.47 ± 4.00 | 109.18 ± 14.90 | 31.84 ± 1.2 | 14.83 ± 3.34 | 84.23 ± 15.91 | 114.21 ± 12.27 | 93.19 ± 4.21 | 0.9 ± 0.08 | 2.4 ± 0.41 | 61.72 ± 5.51 | 45.66 ± 3.46 |
|  | Mesh ID 8 | 649.89 ± 30.79 | 133.62 ± 4.49 | 55.22 ± 3.20 | 8.60 ± 1.03 | 50.40 ± 12.67 | 79.31 ± 1.98 | 118.60 ± 4.08 | 65.57 ± 4.60 | 1.00 ± 0.04 | 4.85 ± 0.45 | 53.01 ± 6.35 | 63.13 ± 3.88 |
|  | Mesh ID 10 | 475.67 ± 27.33 | 89.55 ± 7.89 | 64.36 ± 11.15 | 17.20 ± 8.64 | 45.00 ± 3.29 | 63.78 ± 3.19 | 96.43 ± 14.42 | 102.01 ± 2.67 | 4.07 ± 0.32 | 4.37 ± 0.71 | 57.29 ± 0.95 | 63.82 ± 4.96 |

# Table S2: Morphological parameters for the nine meshes.

|  |  | TP [%] | EP [%] | TP [g/m^2^] | T [mm] | FA_w_ [°] | tanFA_w_ | PS_w_ |
| --- | --- | --- | --- | --- | --- | --- | --- | --- |
|  |  |  |  |  |  |  |  |  |
| **LW** | Mesh ID 1 | 67.20 | 56.28 | 40.34 | 0.49 | 80.16 | 5.76 | 3.04 |
|  | Mesh ID 2 | 65.98 | 61.45 | 48.61 | 0.53 | 27.52 | 0.52 | 1.56 |
|  | Mesh ID 3 | 63.50 | 52.73 | 45.74 | 0.45 | 69.44 | 2.67 | 2.32 |
|  | Mesh ID 4 | 69.37 | 67.45 | 35.53 | 0.37 | 81.20 | 6.46 | 1.71 |
|  | Mesh ID 5 | 70.41 | 54.36 | 44.64 | 0.55 | 39.94 | 0.84 | 2.43 |
|  | Mesh ID 6 | 66.09 | 54.99 | 50.38 | 0.62 | 45.56 | 1.02 | 2.41 |
| **SW** | Mesh ID 7 | 42.08 | 0 | 110.55 | 0.71 | 63.81 | 2.03 | 1.75 |
|  | Mesh ID 8 | 49.83 | 0 | 76.79 | 0.52 | 25.61 | 0.48 | 1.93 |
|  | Mesh ID 10 | 37.62 | 0 | 132.41 | 0.56 | 30.10 | 0.58 | 1.80 |
